# Use of Protoporphyrin Fluorescence to Determine Clinical Target Volume for Non-melanotic Skin Cancers Treated with Primary Radiotherapy

**DOI:** 10.7759/cureus.767

**Published:** 2016-09-04

**Authors:** Stephanie Casey, Lara Best, Olga Vujovic, Kevin Jordan, Barbara Fisher, Deborah Carey, Deborah Bourdeau, Edward Yu

**Affiliations:** 1 London Regional Cancer Program, London Health Sciences Center; 2 Department of Radiation Oncology, Nova Scotia Cancer Center; 3 Department of Radiation Oncology, London Regional Cancer Program, Western University, London, Ontario, CA; 4 Physics, London Regional Cancer Program, Western University, London, Ontario, CA; 5 Department of Medical Biophysics, Western University, London, Ontario, CA; 6 Department of Physics, London Regional Cancer Program, Western University, London, Ontario, CA; 7 Schulich School of Medicine & Dentistry, Western University, London, Ontario, CA

**Keywords:** photo-delineation, ala (δ-aminolevulinic acid), mal (methyl-5-aminolevulinate), protoporphyrin-9 (pp-ix), ctv (clinical target volume), radiotherapy, scc (squamous cell cancer), bcc (basal cell cancer), poorly defined skin cancer

## Abstract

**Purpose:**

Non-melanotic skin cancers remain the most commonly diagnosed cancers. Radiotherapy and surgery are the most common treatment options. Radiotherapy has a recurrence rate of up to 20% for basal or squamous cell cancers. One of the difficulties is to determine the extent of disease for poorly demarcated tumors. This study utilizes protoporphyrin (PpIX) fluorescence to provide information on the extent of subclinical disease for poorly demarcated tumors treated with radiotherapy.

**Materials and Methods:**

For 33 patients, PpIX photo-delineation was used to determine the clinical target volume (CTV2), which was compared to current conventional margins used to account for microscopic disease.

**Results:**

The use of PpIX photo-delineation demonstrated a significantly larger CTV of 15 mm compared to the conventional 10 mm (p = 0.03) for poorly demarcated lesions. A larger CTV was also demonstrated with PpIX photo-delineation for all basal cell carcinomas (13 mm, p = 0.03) as well as for non-nasal lesions (14 mm, p = 0.04). A trend towards an increased CTV was also noted for squamous cell carcinomas (16 mm, p = 0.19) and nasal primary sites (14 mm, p = 0.11). Nasal primary malignancies had multifocal PpIX uptake in 94% of cases. There was one case of local recurrence and one case of distant recurrence, with an average follow-up time of 22 months.

**Conclusions:**

The margins currently used to account for subclinical disease may underestimate the extent of microscopic spread for poorly demarcated tumors. Longer follow-up with larger pools of patients are necessary to determine if using PpIX photo-delineation translates into significantly improved clinical outcomes.

## Introduction

Squamous cell carcinoma (SCC) and basal cell carcinoma (BCC) of the skin continue to be the most commonly diagnosed cancers in North America and worldwide [1]. The majority of skin cancers occur in sun exposed areas such as the head and neck. SCC has a higher propensity for lymphatic spread and metastases in later stages, while BCC tends to be locally destructive. There are multiple treatment modalities for cancer management, including radiotherapy, surgery, topical chemotherapy, and ablative techniques.

Radiotherapy provides local control of approximately 90–95% and 80–85%, respectively, for small and large BCCs [2]. The local control rates of SCCs treated with radiation vary; however, most studies suggest local control is similar to surgery with long-term local control between 80–90% [2-3]. The challenge of radiotherapy is determining the subclinical extent of disease to determine adequate treatment volumes. With surgical excision it is possible to pathologically assess margin status. However, there is no method to ensure inclusion of the cancer in radiotherapy treatment fields. Thus, for poorly demarcated tumor borders, a large clinical target volume or margin beyond the gross visible disease—used to account for microscopic spread—is required to ensure adequate coverage of subclinical disease. The margin conventionally used is 10 mm. Even so, the recurrence rate after radical radiotherapy is approximately 10–20%. This suggests that outcomes may be improved by a method that aids in better tumor delineation, particularly for sclerosing and poorly demarcated tumors. One proposed method would be to employ a material that is preferentially taken up by tumor cells and easily visualized, such as the fluorescence via photosensitization.

Photodynamic therapy (PDT) involves introducing a prodrug, such as δ-aminolevulinic acid (ALA) or methyl-aminolevulinic acid (MAL) dissolved in a cream, to a cutaneous malignancy and the surrounding area. The ALA or MAL molecules are preferentially taken up by abnormal cells such as skin cancers. MAL is converted to ALA during cellular uptake. In the mitochondria, ALA is then converted to protoporphyrin IX (PpIX), a metabolite in the heme synthesis pathway. The molecules are excited to a higher energy state by illumination with blue or red light. The release of energy to return the PpIX to its ground state can occur in one of two different pathways, both of which occur simultaneously [4-5]. The first pathway for the released energy when PpIX returns to its ground state is non-radiative transfer to singlet oxygen, which leads to a cascade of events culminating in cell damage and apoptosis. There is evidence for its use in treatment of superficial non-melanotic skin cancers such as BCCs and SCCs, actinic keratosis, and Bowen’s disease with a local control rate of 70–100% [5-6]. The second pathway, usually associated with the application of blue light, involves energy released in the form of red fluorescent light. Since the ALA is preferentially taken up by skin cancers, the borders of the tumor can be readily visualized as they will fluoresce brighter than the background uptake.

The primary objective of this study was to prospectively enroll non-melanotic skin cancer patients to determine if using PpIX fluorescence for photo-delineation of subclinical disease leads to a significant difference in the CTV as compared to the commonly used margin of 10 mm for poorly demarcated tumors. A secondary end point was to determine if the use of PpIX fluorescence to determine treatment borders leads to better local control compared to historical results with radiation alone.

## Materials and methods

Ethics approval was obtained through the Health Sciences Research Ethics board. Between May 2011 and June 2014, 33 patients with biopsy-confirmed basal cell or squamous cell carcinomas were prospectively enrolled in the study. The lesions had to be readily visible. Of the 33 lesions, seven were included as a control group as they demonstrated well demarcated borders with no evidence of satellitosis.

The patients were brought to the clinic and their lesions and cervical lymph nodes were assessed. Three ink marks were placed on the skin each 5 cm apart from each other for use as fiducial markers and for scale. A transparency slide was placed over the lesion and the three fiducial marks were transcribed onto it. The physician marked the gross tumor volume (GTV) as seen under normal white light on the transparency. Digital photographs were taken using a Nikon D50 camera with a linearly polarized flash and a linear polarizer at the lens. Autofluorescence (specifically, light emitted by skin cells without the addition of ALA) was excited by a filtered actinic lamp with a peak wavelength of 415 nm, and the images were recorded using a yellow #2 glass filter.

**Figure 1:**
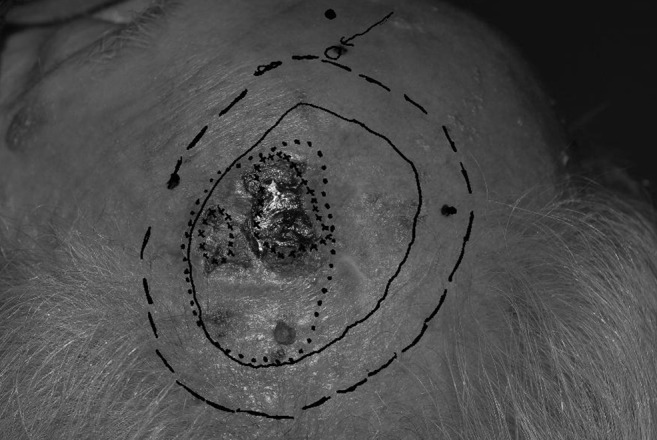
Squamous cell carcinoma. Treatment borders post-PpIX fluorescence Dotted line: GTV Solid line: CTV2 Hash line: edge of the radiotherapy field border for treatment *: Study CTV measurement used for comparison

δ-aminolevulinic acid (ALA) infused glaxal base (10% by weight) or Metvix (methyl-5-aminolevulinic acid, MAL) diluted with equal mass glaxal base was applied to the lesion and surrounding area and then covered with an occlusive Tegaderm dressing. Approximately half the patients were examined using ALA, the remainder with MAL. After two hours the excess cream was removed. A new transparency was placed over the lesion and the three fiducial markers were transcribed. The filtered actinic light was shone on the lesion in a dark room using a peak wavelength of 415 nm for excitation, and the physician, wearing yellow tinted glasses, marked the fluorescing area corresponding to gross and microscopic disease onto the transparency, as is seen in Figure 1. 

**Figure 2:**
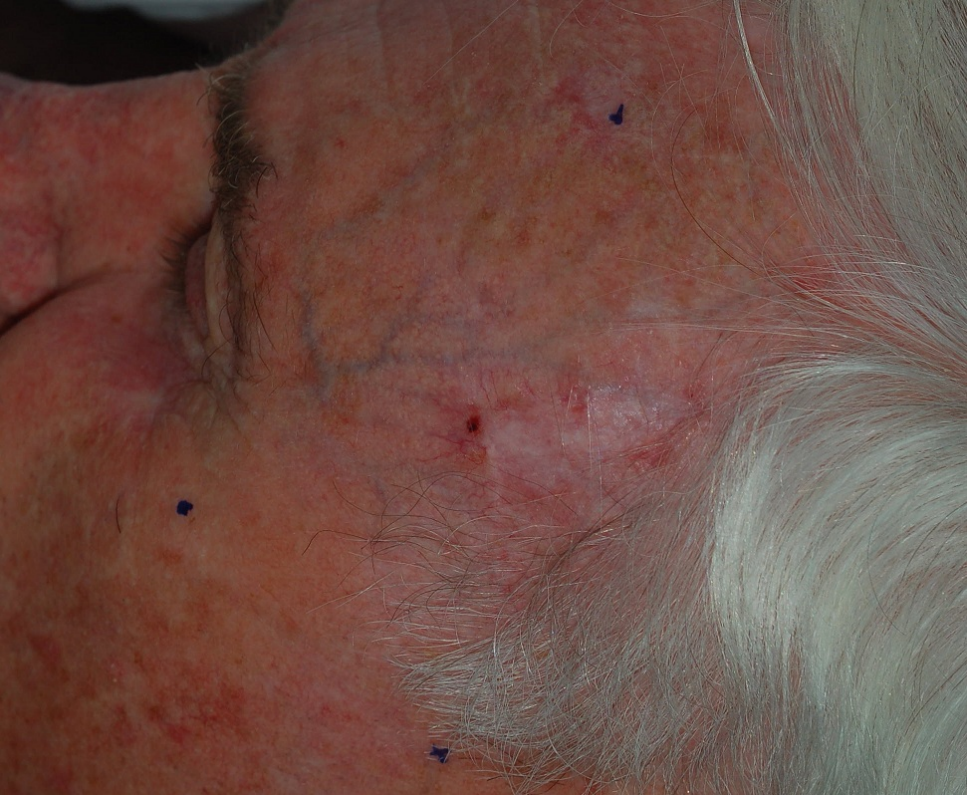
Sclerosing squamous cell carcinoma of forehead Visualized under white light.

**Figure 3:**
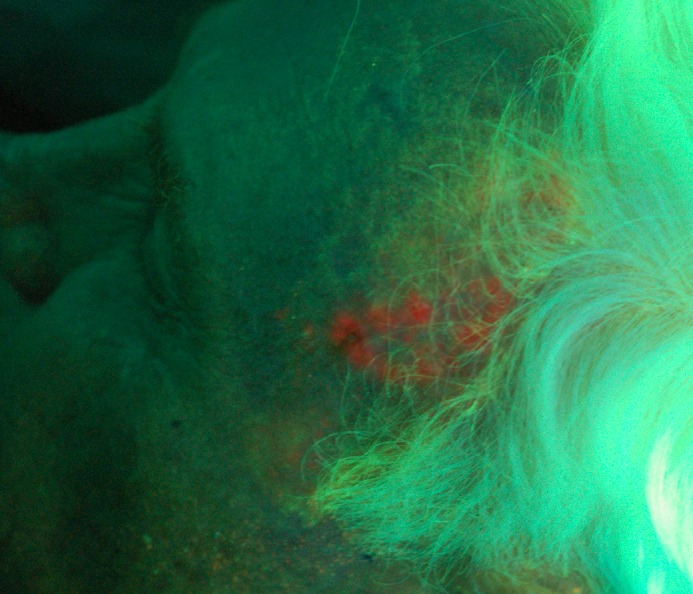
Sclerosing squamous cell carcinoma of forehead Depicted with protoporphyrin IX fluorescence.

The area of fluorescence due to PpIX is denoted as the trial clinical target volume (CTV2). It is important to note that this area accounts for fluorescence due to PpIX as seen on the skin surface and does not give information regarding the potential depth of the malignancy. These values also represent an average expansion from the gross disease. The region of PpIX fluorescence did not uniformly expand around the gross disease, and an average number to represent the expansion superiorly, inferiorly, and laterally at the skin surface was used for simplification. An example of a poorly defined squamous cell carcinoma is seen in Figure 2 (white light), and the corresponding lesion with a larger region of multifocal uptake is observed via PpIX fluorescence in Figure 3.

**Figure 4:**
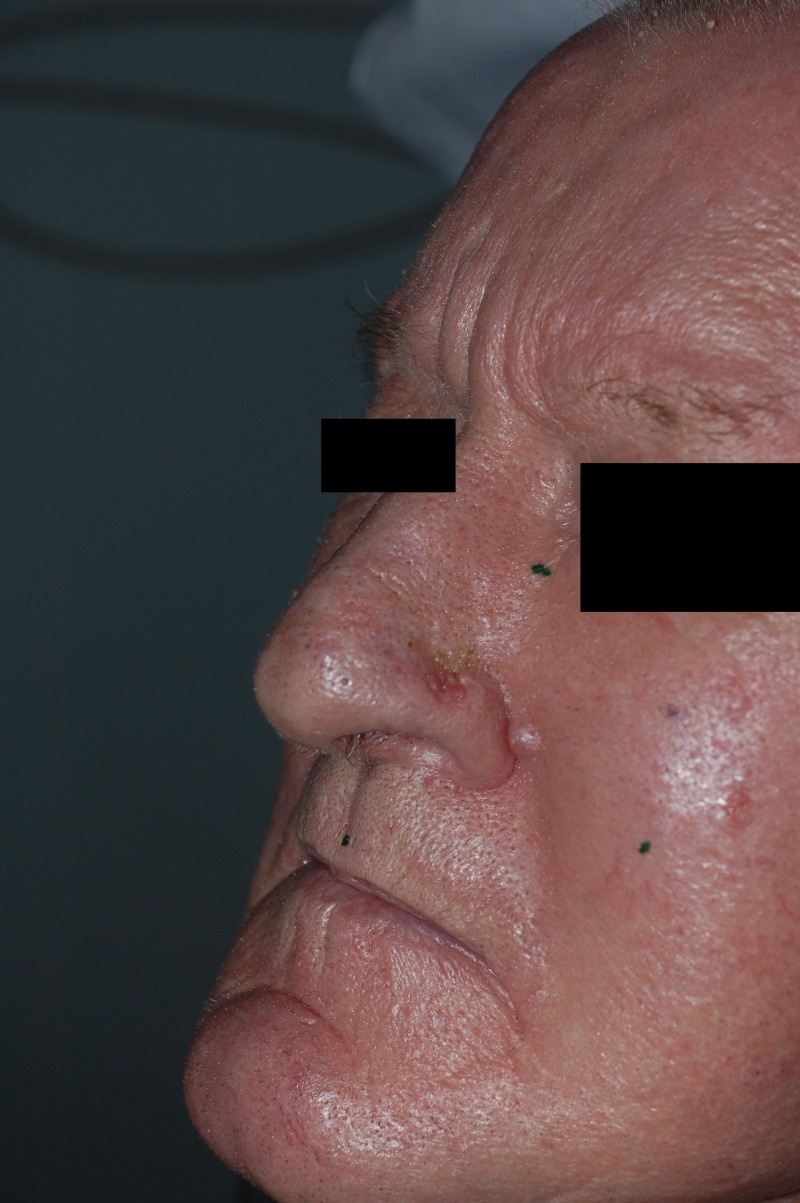
Basal cell carcinoma adjacent to the nostril Viewed under white light.

**Figure 5:**
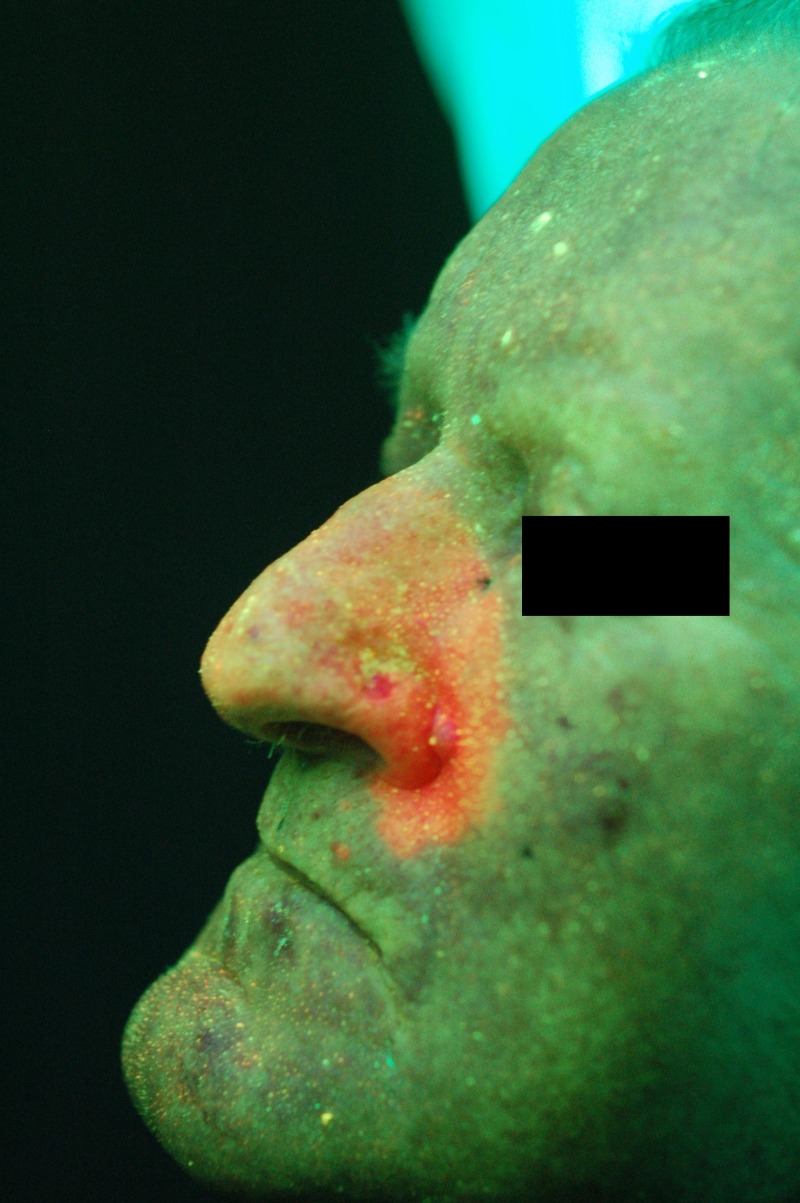
Basal cell carcinoma adjacent to the nostril Depicted with protoporphyrin IX fluorescence.

Figure 4 and Figure 5 show a BCC. Digital photographs of the transparencies with a ruler placed on top for size reference were taken for image analysis. The final volume treated has a margin of 2–3 mm around the CTV2 that took into account daily patient set-up errors and the characteristics of the treatment beam used.

Radiotherapy treatment was given using 4 or 6 MV photons, an appositional 9–12 MeV electron beam or direct 100 kVp photons. The radiation oncologist decided on the treatment modality based on the patient and tumor characteristics. Kilovoltage and megavoltage photons were prescribed to Dmax (the depth of maximum dose), while electrons were prescribed to the 90% isodose line at depth. For the photon and electron treatment, bolus (supraflab or wax) was placed over the lesion to ensure adequate skin dosage. All tumors were treated to 50 Gray (Gy) in 20 once-daily fractions. The only exception was a fast growing squamous cell carcinoma post-excision that was treated to 60 Gy in 30 once-daily fractions. The patients were reviewed two-three months after the completion of therapy and every six to 12 months afterwards.

A two-tailed one sample t-test was used for statistical analysis to determine if the observed PpIX photo-delineation of CTV2 was significantly larger than the conventionally used margin to account for subclinical disease. The conventional clinical target volumes used for comparison were 5 mm or 10 mm for well or poorly demarcated tumors, respectively. Significant results required a p-value ≤0.05. For comparison purposes, the reported CTV2 was the maximum distance from the edge of the GTV to the edge of the fluorescing area, as the fluorescing area was rarely uniform around the GTV. These values were compared to a conventionally used CTV of 5 mm or 10 mm for well or poorly demarcated tumors, respectively. The control group (well demarcated tumors) and experimental group (poorly demarcated tumors) were analyzed separately. A subgroup analysis was used to determine if PpIX photo-delineation was of most use for detecting microscopic disease in BCCs, SCCs, nasal or non-nasal primary sites.

## Results

Thirty-three patients were enrolled. The demographic and treatment data for all enrolled patients are seen in Table 1.

**Table 1:** Patient demographics and treatment

Patient Characteristics	Control Group	Experimental Group
Number of lesions	7	26
Age, years (median)	80 (68 – 90)	81 (47 – 91)
Number male (%)	4 (57%)	17 (65%)
BCCs, metaplastic	7 (100%)	23 (88%)
SCCs	0 (0%)	3 (12%)
4 – 6 MV photon treatment	3 (43%)	13 (50%)
9 – 12 MeV electron treatment	2 (29%)	8 (31%)
100 kVp treatment	2 (29%)	5 (19%)

The only difference between the control and experimental groups was that the control group had tumors with well-defined tumor borders. These included nodular lesions or rodent ulcers with heaped up or rolled borders. The experimental group included any tumors that were post subtotal biopsy, sclerosing, or infiltrative with hard-to-define borders. All lesions were staged according to the American Joint Committee on Cancer (AJCC) 2010 staging system [1]. Thirty of the lesions were T1, three were staged as T2, and all had clinically negative lymph nodes.

The average CTV2 for the groups are shown in Table 2.

**Table 2:** Comparison of standard versus PpIX fluorescent margins to account for subclinical disease

	Control Group (mm)	Experimental Group (mm)
Conventional CTV, mm	5	10
Trial CTV (CTV2), mm	9 (0 – 40)	15 (0 – 41)
P-value	p = 0,50	p = 0,01
Percentage lesions not adequately covered (CTV2 > CTV1)	29%	62%

For the control and experimental groups, a conventional CTV of 5 mm and 10 mm respectively, was compared to the maximum PpIX fluorescence distance from the GTV (CTV2). The difference was not statistically significant for the control group. For the experimental group, the average CTV was significantly larger using PpIX photo-delineation, 15 mm versus 10 mm (p = 0.03) for poorly demarcated lesions.

Two of the seven well-demarcated tumors had fluorescence beyond 5 mm. However, one of these was in a patient with extensive sun-related actinic changes. This is in contrast to the poorly demarcated tumors, where 16 of the 26 patients (62%) had microscopic disease, as evidenced by PpIX fluorescence, beyond the expected 10 mm. In this group, 12 (46%) had disease beyond 15 mm from the visible gross tumor volume. Figure 3 and Figure 5 provide representative examples of how the region of PpIX fluorescence exceeds that which would have been located inside a standard CTV.

Three patients with SCC had an average maximum CTV2 distance of 16 mm. This approached statistical significance compared to the conventional CTV expansion of 10 mm (p = 0.19). The remaining 30 lesions were BCCs or metaplastic carcinomas (one patient) with an average maximum fluorescent distance of 13 mm (p = 0.03). Of the sites treated, 17 (51%) involved the nose. The average CTV2 for nasal lesions was 14 mm, but this was not significantly different from the conventional CTV (p = 0.11). For non-nose sites the average maximum fluorescent distance of 14 mm was statistically significant (p = 0.04). Of note, 94% of the patients treated for a nasal primary demonstrated multifocal disease with photo-delineation compared to 44% at non-nasal sites.

All patients were scheduled for assessment two to three months post-treatment then semiannually. The average follow-up was 22 months with a range of 0–44 months. Two patients were lost to follow-up. There was one local relapse in a 90-year-old male with a T2N0 pre-auricular metatypical cancer with well demarcated borders. PpIX fluorescence was not seen outside of the lesion, suggesting no satellitosis (CTV2 = 0 mm expansion on the visible disease). His treatment volume included CTV2 + 15 mm. He was treated using 9 MeV direct electrons to 50 Gy in 20 fractions with 1 cm of bolus over the lesion. He had an in-field recurrence at 11 months, which was successfully salvaged with surgery. Pathology revealed a 5-mm BCC.

One distant relapse occurred in a male patient treated post-resection for a rapidly progressive T2N0 SCC of the anterior chest. He was treated aggressively to 60 Gy in 30 fractions using 9 MeV electrons and a 1-cm bolus. PpIX fluorescence was seen 20 mm away from the visible disease (CTV2). His treated volume included CTV2 + 10 mm. At one month he was noted to have left axillary adenopathy. At four months post-treatment he had bilateral axillary, lung, and splenic metastases. He died seven months after the completion of radiotherapy.

## Discussion

Non-melanotic skin cancers are the most commonly diagnosed cancers, and effective and cost-efficient methods of treatment of these cancers are important. Proper management is important as local control diminishes by approximately 10% each time the cancer recurs and the risk of metastatic disease increases [2-3]. One of the main difficulties is determining the margins for these tumors, leading to positive margins at surgery, or marginal or local recurrences after radiotherapy. PpIX photo-delineation is one method that has been studied in the surgical domain for prediction of surgical margins. Published data has demonstrated that using PpIX for photo-delineation for non-melanotic cutaneous malignancies leads to lower rates of pathologically-positive margins compared to surgery alone [4, 7].

There is no published data using PpIX photo-delineation in non-melanotic skin cancers for determining treatment volumes for external beam radiotherapy. By extrapolating from surgical data, it is expected that the use of PpIX for photo-delineation will provide more determination of tumor extent for poorly defined skin cancers, leading to better local control [4, 7].

Table 3 summarizes several patient studies that have used PpIX fluorescence to detect skin lesions, delineate tumor borders, or evaluate treatment response.

**Table 3:** Summary of PpIX fluorescence delineation studies Debridement = (?) if not specified, CCC = red/green/blue digital camera, eye = visual examination of fluorescence, point measurement. Numbers in brackets next to the author name indicate the reference number.

Year	Author	# PpIX Lesions	Debride	Cream	Incubation Time (hrs)	Wavelength (nm)	Detector
1995	Martin [13]	17	?	ALA 20%	3 to 18	400-500 (Xe flash)	CCD
1999	Golub [24]	14	no	ALA 10 to 30%	0 to 48	410	spectrometer
2001	Na [28]	21	?	Metvix	4	370	spectrometer
2003	Ericson [11]	23	no	ALA 20%	1 to 4	365	CCD
2005	Angell-Petersen [12]	50	yes	Metvix	2 to 3	407	point
2006	Stenquist [25]	12	?	ALA 20%	3	365	CCD
2007	Brandt [4]	33	no	ALA 5%	3	365,415	eye, CCD (red)
2007	Won [14]	50	alcohol	Metvix	3	365	red
2008	Gambichler [26]	16	?	ALA 20%	3	370-440 (Xe)	CCD
2009	de Leeuw [27]	93	acetone	ALA-liposome	2	405 (LED, pulsed)	CCD
2009	Navickiene [15]	164	?	ALA vs Metvix	2 to 4	401-405	CCD
2010	Lee [5]	142	?	ALA 20%	6	365	eye, CCD (red)
2010	Wetzig [16]	22	saline	Metvix	3	405 (LED, pulsed)	CCD
2011	Sandberg [9]	35	yes	Metvix	3	365	CCD
2011	Tierney [7]	20	yes	Metvix	13	365	CCD
2012	van der Beek [10]	30	no	ALA-liposome	2.5	405 (LED, pulsed)	CCD
2013	Jeon [17]	38	yes	ALA 20%, Metvix	3, 6	365	eye, CCD (red)
2014	Jeon [18]	59	yes	ALA 20%	6	365	eye, CCD (red)
2014	Andrade [8]	71	no	ALA solution	0.2 - 1	380 - 420	CCD
2014	Truchelo [20]	29	?	Metvix	3	400 (LD flash)	eye, CCD
2015	This study	33	no	ALA 5%, Metvix 50%	2	415	eye, CCD (red)

The studies are arranged chronologically to highlight the introductions of technical advances such as Metvix, liposomal ALA formulation, high power violet LEDs (light emitting diode), and photographic-grade digital cameras. The average number of lesions in these studies was 46. Patients with a small number of isolated, well-defined lesions generally do not require delineation methods beyond clinical examination. Consistent lesion debridement is necessary for the comparison of PDT studies, autofluorescence, and the relative intensity of the PpIX fluorescence but is not essential for delineation measurements [8]. Metvix was found to be superior to ALA for BCC delineation due to a greater PpIX fluorescence intensity, though other studies have found ALA formulations also to be effective [9-10]. Topical incubation times varied; in general, a maximum PpIX fluorescence in lesions is observed with four- to six-hour incubations when probing with light in the 360–500 nm wavelength range. In studies, including this one, which use PpIX fluorescence to determine if it provides a clinical benefit in lesion delineation, short incubation times were necessary for practical use of the technique and were not noted to compromise delineation findings [11-12]. However, if information on the depth of penetration was desired, longer incubation times would be required [13].

A few studies have been published comparing the use of PpIX fluorescence for photo-delineation of tumor borders with the final surgical pathology. Brandt found that using PpIX fluorescence led to clear pathological resection margins while surgeon-determined margins required re-excision in nine percent of cases [4]. Tierney completed a similar trial comparing MAL fluorescence volumes with post-Mohs surgical defect volumes [7]. The area of fluorescence correlated highly with the post-Mohs defect size. Won used fluorescent image analysis after application of ALA or MAL to facial BCCs to determine tumor area [14]. Gross disease was removed and biopsies were taken from the suspected areas of involvement (as indicated by areas of fluorescence under blue light) as well as from areas thought to be normal tissue. Tumor cells were found in 80% and five percent of fluorescing and non-fluorescing areas, respectively. The sensitivity and specificity of photo-delineation was found to be 94% and 83%, respectively. Navickiene looked at malignant skin lesions using either ALA or MAL photo-delineation for surgical margin status [15]. He found that ALA or MAL provided a tumor margin sensitivity of 95%, a specificity of 89%, a positive predictive value of 86%, and a negative predictive value of 96%. Most studies demonstrated a strong correlation between subclinical tumor margin and PpIX fluorescence and provided evidence for its use in determining tumor borders for poorly demarcated tumors as opposed to well-delineated or deeply pigmented lesions [16-19].

The greatest benefit of PpIX photo-delineation was for poorly defined tumors, which are historically difficult to delineate under normal white light. Photo-delineation provided a statistically significant increase in the CTV compared to conventional margins for subclinical disease. In our data set, 62% of poorly demarcated tumors would not have been adequately covered by the conventional CTV of 10 mm. This finding indicates the reason for the higher local recurrence rate seen with external beam radiotherapy versus surgical excision is the larger margins. However, our follow-up is not yet sufficiently long to determine if the statistical significance noted for the use of PpIX fluorescence to determine subclinical disease will translate into a clinically significant difference, such as a lower local recurrence rate. The only benefit of photo-delineation for well-defined or nodular lesions is to identify satellite lesions.

Again, it is important to note that the region of PpIX fluorescence did not extend uniformly around the GTV. The pattern of fluorescence tended to be serpiginous, or at least more significant in one direction. As such, the use of photo-delineation can help mould radiation to include the subclinical disease, allowing sparing of cancer-free areas that would normally be covered by a uniform CTV. This would be of particular use in areas close to critical structures such as the eye.

Further analysis of the data is hindered by the small sample size. Thus a statistical benefit for this method of tumor delineation was not reached for either SCCs or nasal lesions in subgroup analysis. Interestingly, non-nasal primary sites showed statistically significant benefit for photo-delineation with a small sample size of seven patients, while there is a trend, though not statistically significant, for nasal primaries. This contrasts to the often multifocal disease noted for nasal primaries (94%) versus non-nasal primaries (44%). The finding may be due to the nose being a relatively small area, thus most satellite lesions on the nose are contained within the 1-cm CTV expansion. Also, the nose is a common site of malignancy due to its higher exposure to sunlight, lending itself to being more prone to field cancerization and development of a second malignancy. Clinical experience at our center has been that skin malignancies that occur on the nose generally have multifocal uptake when PpIX fluorescence is used.

Another limitation is the lack of biopsy results attained for the multifocal disease suggested by the PpIX fluorescence. A benign disease, such as actinic keratosis is also known to fluoresce with the application of ALA or MAL. Thus, while we treated all fluorescing areas as potential microscopic spread, pathological confirmation was not available. That said, previous data has shown that PpIX fluorescence has a high sensitivity and specificity for invasive disease [14-15, 20]. A recent publication also noted that PpIX fluorescence was generally less significant with actinic keratoses because of the increased layer of keratin—this contrasted with the greater uptake of ALA and subsequent PpIX fluorescence observed with BCCs [8]. In this study keratin was not removed since delineation and not treatment was the primary goal.

Additionally, biopsying all sites of fluorescence could be technically challenging and disfiguring, especially in areas with limited excess skin such as the nose [21]. Our decision to treat the entire nose when multifocal disease was observed is supported by pathological data suggesting that nasal primaries are at a particularly higher risk of having extensive subclinical disease [22-23]. It has also been established that the nose is at a higher risk for recurrence compared to other body sites, suggesting that it may be prudent to treat it aggressively as recurrent disease can be difficult to treat [21]. Because the number of unifocal nasal lesions was so few, it is doubtful that even a study with larger numbers could demonstrate a significant percentage of patients with unifocal fluorescence.

Patients with large areas of sun damage may pose another challenge in that the results from use of PpIX fluorescence for photo-delineation may be muted. This can be problematic in patients with significant sun damage adjacent to a malignancy as it lowers the contrast between the malignancy and normal skin when PpIX fluorescence is utilized. This issue is also seen with using autofluorescence to help determine sites of subclinical disease. However, because of the higher concentrations of PpIX present in abnormal cells due to the uptake of MAL and ALA, in addition to the amounts of PpIX that the cells themselves make, the ratio of fluorescence observed is much greater when ALA and MAL preparations are used.

Another area of difficulty in assessing tumor margins is that the amount of fluorescence from malignant lesions may vary, as can the ratio of fluorescence between the lesion and the normal epidermis [13, 24]. This could have led to errors in deciding whether fluorescence represented disease versus background epidermal fluorescence. The ratio of red PpIX fluorescence to either blue or green autofluorescence can provide images with higher contrast by normalizing for differences in excitation intensity and emission collection efficiency across the images [8-11, 16, 25-28]. The image ratio approach will be most helpful in making clinical decisions if it is available for real time evaluation of the patient’s lesions. This clinical study was designed to use only the physician’s visual interpretation of the PpIX fluorescence of the lesion for delineation. The intent was to investigate the value of adding PpIX fluorescence to standard white light examinations. Selected patient images were later examined to compare red/green images of the autofluorescence and PpIX fluorescence. It was found that obvious lesions in the PpIX image typically had red:green ratios in the 1.2 to 1.3 range. This ratio is lower than the threshold ratio of 1.4 previously reported likely due to the shorter incubation time, lower ALA concentration, and lack of lesion debridement [11, 26]. Qualitatively, the red:green ratio images showed more contrast than the original images. However, after observing several sets of autofluorescence and PpIX fluorescence images, as well as the same photos with a red:green ratio applied, the same features were seen in both sets. The effect of self-training indicates that examining sets of images acquired with the same protocol allows greater confidence in evaluating additional images acquired by same method. Moreover, a comparison of autofluorescence and PpIX fluorescence images showed higher sensitivity with PpIX fluorescence. It appears that more information improves sensitivity and accuracy and is justified for lesions where accurate determination of borders is critical (i.e. near nerves and ducts) [10].

Blue light excitation has the advantage of providing a composite image of superficial autofluorescence and PpIX fluorescence. However, there is no direct information regarding a lesion’s border at depths below approximately 0.1 mm. Red light excitation is an option to probe PpIX at greater depths. However, electronic detection would be required since the human eye is relatively insensitive to red light, and laser excitation is needed because of the lower absorption of PpIX and signal attenuation by overlying tissue. Additionally, light scattering by the tissue would further degrade the image’s detail. An alternative option may involve first performing a red light PDT on the area of interest and then observing the inflammatory response [29]. This may be a practical approach with the added clinical benefit of treating a superficial disease while delineating the lesion for subsequent radiotherapy.

Luluel Khan and colleagues demonstrated a need for larger margins for microscopic disease when performing clinical markups for skin malignancies using white light [30]. They noted a relationship between histology, size of the tumor, and the margins required to properly account for microscopic extension, which correlated with a need for re-excision. Their recommendations for larger CTVs for SCCs or ill-defined or large BCCs compare favorably with our findings.

One patient died of disease; however, the death is not thought to be related to suboptimal treatment. At the time of treatment of his anterior chest disease he already demonstrated evidence of aggressive disease, having undergone three previous resections with the last recurrence occurring three months after resection with negative margins. He did not have a metastatic workup prior to radiotherapy, but a computed tomography (CT) scan of the thorax performed within one month of treatment completion demonstrated large left-sided axillary adenopathy with necrotic centers. This suggests that he may have had at least regional disease prior to initiating radiotherapy. Also, his original site of disease remained free of recurrence until he died seven months after the treatment was complete. Another patient had a recurrence within the high dose region despite receiving appropriate dose to the area. This was salvaged successfully with surgery.

## Conclusions

Overall, our data suggests that the implementation of PpIX photo-delineation is of benefit in determining the extent of subclinical disease, especially for poorly defined tumors. This is evidenced by the statistically significant findings noted for CTVs determined with photo-delineation for BCCs and non-nasal lesions. The photo-delineation use is associated with a local control rate of 93.9% at 22 months. In the case of SCCs and nasal lesions, trends were noted for larger CTVs, and as such, a larger sample size and longer follow-up are required to determine if photo-delineation is useful in such situations.
